# Supplementary material for: Conditional deletion of glucocorticoid receptors in rat brain results in sex-specific deficits in fear and coping behaviors
Source: eLife. 2019 Jul 22;8:e44672. doi: 10.7554/eLife.44672 (PMC6645713; doi:10.7554/eLife.44672)
Supplement: Supplementary file 1. [file elife-44672-supp1.pptx]

## Slide 1
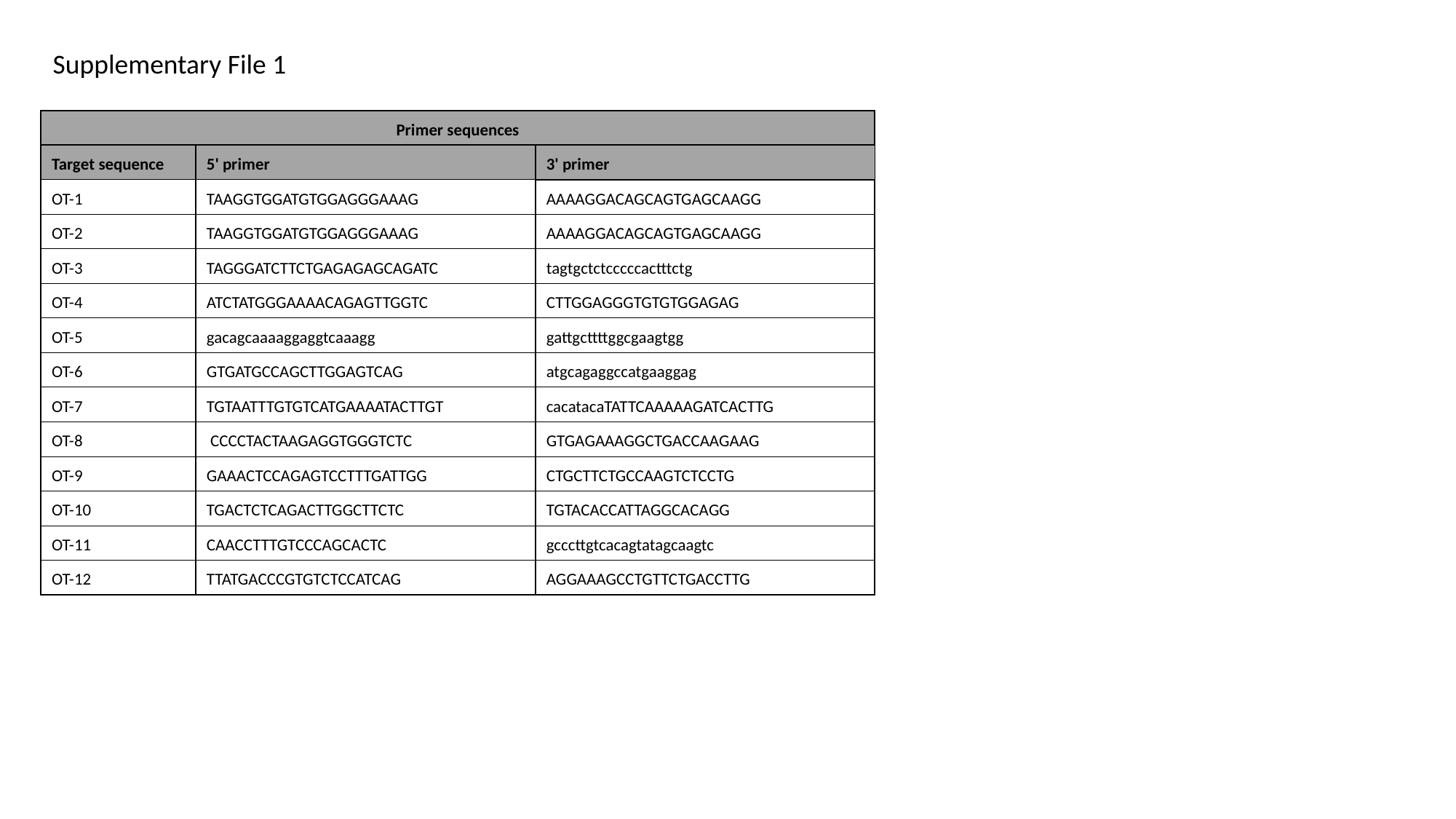

Supplementary File 1
| Primer sequences | | |
| --- | --- | --- |
| Target sequence | 5' primer | 3' primer |
| OT-1 | TAAGGTGGATGTGGAGGGAAAG | AAAAGGACAGCAGTGAGCAAGG |
| OT-2 | TAAGGTGGATGTGGAGGGAAAG | AAAAGGACAGCAGTGAGCAAGG |
| OT-3 | TAGGGATCTTCTGAGAGAGCAGATC | tagtgctctcccccactttctg |
| OT-4 | ATCTATGGGAAAACAGAGTTGGTC | CTTGGAGGGTGTGTGGAGAG |
| OT-5 | gacagcaaaaggaggtcaaagg | gattgcttttggcgaagtgg |
| OT-6 | GTGATGCCAGCTTGGAGTCAG | atgcagaggccatgaaggag |
| OT-7 | TGTAATTTGTGTCATGAAAATACTTGT | cacatacaTATTCAAAAAGATCACTTG |
| OT-8 | CCCCTACTAAGAGGTGGGTCTC | GTGAGAAAGGCTGACCAAGAAG |
| OT-9 | GAAACTCCAGAGTCCTTTGATTGG | CTGCTTCTGCCAAGTCTCCTG |
| OT-10 | TGACTCTCAGACTTGGCTTCTC | TGTACACCATTAGGCACAGG |
| OT-11 | CAACCTTTGTCCCAGCACTC | gcccttgtcacagtatagcaagtc |
| OT-12 | TTATGACCCGTGTCTCCATCAG | AGGAAAGCCTGTTCTGACCTTG |
