# Supplementary material for: Conditional deletion of glucocorticoid receptors in rat brain results in sex-specific deficits in fear and coping behaviors
Source: eLife. 2019 Jul 22;8:e44672. doi: 10.7554/eLife.44672 (PMC6645713; doi:10.7554/eLife.44672)
Supplement: Transparent reporting form [file elife-44672-transrepform.docx]

***eLife’s* transparent reporting form**

We encourage authors to provide detailed information *within their submission* to facilitate the interpretation and replication of experiments. Authors can upload supporting documentation to indicate the use of appropriate reporting guidelines for health-related research (see [EQUATOR Network](http://www.equator-network.org/%20)), life science research (see the [BioSharing Information Resource](https://biosharing.org/)), or the [ARRIVE guidelines](http://www.plosbiology.org/article/info:doi/10.1371/journal.pbio.1000412) for reporting work involving animal research. Where applicable, authors should refer to any relevant reporting standards documents in this form.

If you have any questions, please consult our Journal Policies and/or contact us: [editorial@elifesciences.org](mailto:editorial@elifesciences.org).

**Sample-size estimation**

- You should state whether an appropriate sample size was computed when the study was being designed
- You should state the statistical method of sample size computation and any required assumptions
- If no explicit power analysis was used, you should describe how you decided what sample (replicate) size (number) to use

Please outline where this information can be found within the submission (e.g., sections or figure legends), or explain why this information doesn’t apply to your submission:

Methods/statistical analysis (page 15) states sample size determined by number of animals of a certain genotype derived in the cohort. See below for effect size analyses.

**Replicates**

- You should report how often each experiment was performed
- You should include a definition of biological versus technical replication
- The data obtained should be provided and sufficient information should be provided to indicate the number of independent biological and/or technical replicates
- If you encountered any outliers, you should describe how these were handled
- Criteria for exclusion/inclusion of data should be clearly stated
- High-throughput sequence data should be uploaded before submission, with a private link for reviewers provided (these are available from both GEO and ArrayExpress)

Please outline where this information can be found within the submission (e.g., sections or figure legends), or explain why this information doesn’t apply to your submission:

Methods/corticosterone measurements after acute restraint (page 15) for technical replicates in corticosterone assay.

Methods/statistical analysis (page 15) for outliers

No others apply

**Statistical reporting**

- Statistical analysis methods should be described and justified
- Raw data should be presented in figures whenever informative to do so (typically when N per group is less than 10)
- For each experiment, you should identify the statistical tests used, exact values of N, definitions of center, methods of multiple test correction, and dispersion and precision measures (e.g., mean, median, SD, SEM, confidence intervals; and, for the major substantive results, a measure of effect size (e.g., Pearson's r, Cohen's d)
- Report exact p-values wherever possible alongside the summary statistics and 95% confidence intervals. These should be reported for all key questions and not only when the p-value is less than 0.05.

Please outline where this information can be found within the submission (e.g., sections or figure legends), or explain why this information doesn’t apply to your submission:

Statistical analysis described in Methods/Statistical analysis section (page 15).

Group means were used rather than raw data as tests were analyzed with parametric statistical tests.

Exact n values are listed for figure 4 and supplemental file 6 are listed within statistics in Results section.

Statistical software used (Sigma Plot) does not give individual mean/st error/confidence intervals for ANOVAs but can be discerned from graphs in figure 4 and supplemental file 6.

Mean, SEM and confidence intervals are located within the graphs of the results and below:

GRKD females : wt mean = 88.94, SEM = 2.044, n = 10; GRKD mean = 57.77 SEM = 1.828 n =5; 95% confidence interval = -38.07 to -24.2; Cohen’s d = 5.765256

GRKD males : wt mean = 84.96, SEM = 1.29, n = 9; GRKD mean = 67.85 SEM = 2.807 n =6; 95% confidence interval = -23.07 to -11.15; Cohen’s d = 3.066863

Female FC Acquisition: wt mean = 40.784 SEM = 4.038 n = 10; GRKD mean = 57.264 SEM = 5.710 n = 5; p = 0.043

Female FC Extinction: wt mean = 48.174 SEM = 6.497 n = 10; GRKD mean = 64.157 SEM= 9.188 n = 5; p = 0.043

Female FC Extinction recall: wt mean = 32.484 SEM = 7.721 n = 10; GRKD mean = 55.166 SEM = 8.458 n = 5; p = 0.046

Male FC Acquisition: wt mean = 6.739 SEM = 0.49 n = 10; GRKD mean = 6.080 SEM = 0.580 n = 6; p =0.036

Male FC Extinction: wt mean = 64.112 SEM = 6.701 n = 10; GRKD mean = 57.107 SEM = 8.651 n = 6; p = 0.01

Male FC Extinction Recall: wt mean = 56.236 SEM = 5.163 n = 10; GRKD mean = 51.532 SEM 6.665 n = 6; p = 0.014

Male FST immobility Day1: wt mean = 65.167; SEM = 2.051; GRKD mean = 71.003 SEM = 2.199 n = 6; 95% confidence interval = -12.608 to 0.937; Cohen’s d = 0.983

Male FST immobility Day2: wt mean = 65.052 SEM = 2.819 n = 10, GRKD mean = 71.341 SEM = 3.306 n =6; GRKD mean = 428.049 SEM = 21.726 n = 6; 95% confidence interval = -15.849 to 3.269; Cohen’s d = 0.739

Male FST Dives Day 1: wt mean = 6.900 SEM = 0.547 n = 10; GRKD mean = 12.167 SEM = 1.922 n = 6; 95% confidence interval = 1.793 to 8.741; Cohen’s d = 1.64

Male FST Dives Day 2: ; wt mean = 1.100 SEM = 0.407 n = 10; GRKD mean = 5.333 SEM = 1.453 n =6; 95% confidence interval = -6.852 to -1.615; Cohen’s d = 1.747

Female FST immobility Day 1: wt mean = 67.691 SEM = 2.468 n = 10; GRKD mean = 67.221 SEM = 7.168 n =5; 95% confidence interval = -12.558 to 13.497; Cohen’s d = 0.039

Female FST immobility Day 1: wt mean = 68.491 SEM = 4.621 n = 10; GRKD mean = 76.691 SEM = 5.302 n = 5; 95% confidence interval = -24.557 to 8.158; Cohen’s d = 0.619

Female FST day 1 dives: wt mean = 8.6, SEM = 1.607; GRKD mean = 7.294, SEM = 3.262; 95% confidence interval = -7.525 to 6.325; Cohen’s d = 0.096

Female FST day 2 dives: wt mean = 2.346 SEM = 1.077 n = 10; GRKD mean = 6.459 SEM = 3.991 n = 5; 95% confidence interval = -10.864 to 2.637; Cohen’s d =0.304 (data transformed to square root to pass equal variance

Female Cort: wt mean = 510.345 SEM = 34.599 n = 10; GRKD mean = 518.022 SEM = 55.460 n = 5; p = 0.024

Male Cort: wt mean = 305.569 SEM = 18.445 n = 10; GRKD mean = 348.116 SEM = 23.332 n = 6; p = 0.012

(For large datasets, or papers with a very large number of statistical tests, you may upload a single table file with tests, Ns, etc., with reference to sections in the manuscript.)

**Group allocation**

- Indicate how samples were allocated into experimental groups (in the case of clinical studies, please specify allocation to treatment method); if randomization was used, please also state if restricted randomization was applied
- Indicate if masking was used during group allocation, data collection and/or data analysis

Please outline where this information can be found within the submission (e.g., sections or figure legends), or explain why this information doesn’t apply to your submission:

Samples were allocated into experimental groups based on genotype.

Behavioral scoring was done by a research blinded to group – info found in text in methods/statistics (page 15)

**Additional data files (“source data”)**

- We encourage you to upload relevant additional data files, such as numerical data that are represented as a graph in a figure, or as a summary table
- Where provided, these should be in the most useful format, and they can be uploaded as “Source data” files linked to a main figure or table
- Include model definition files including the full list of parameters used
- Include code used for data analysis (e.g., R, MatLab)
- Avoid stating that data files are “available upon request”

N/A

Please indicate the figures or tables for which source data files have been provided:
